# Cost-effectiveness of tailored print communication, telephone motivational interviewing, and a combination of the two: results of an economic evaluation alongside the Vitalum randomized controlled trial

**DOI:** 10.1186/1479-5868-7-64

**Published:** 2010-09-03

**Authors:** Hilde M van Keulen, Judith E Bosmans, Maurits W van Tulder, Johan L Severens, Hein de Vries, Johannes Brug, Ilse Mesters

**Affiliations:** 1 School for Public Health and Primary Care (Caphri), Department of Health Promotion, Maastricht University, the Netherlands; 2 Department of Health Sciences and EMGO Institute for Health and Care Research, VU University Amsterdam, the Netherlands; 3 Institute for Health Policy and Management, Erasmus University Rotterdam, the Netherlands; 4 School for Public Health and Primary Care (Caphri), Department of Health Organization, Policy, and Economics, Maastricht University, Maastricht, the Netherlands; 5 EMGO Institute for Health and Care Research, VU University Medical Centre, Amsterdam, the Netherlands; 6 School for Public Health and Primary Care (Caphri), Department of Epidemiology, Maastricht University, the Netherlands

## Abstract

**Background:**

The aim of the present study was to evaluate the cost-effectiveness of tailored print communication (TPC), telephone motivational interviewing (TMI), a combination of the two, and no intervention on two outcomes in adults aged 45 to 70, half of them having hypertension: increasing the number of public health guidelines met for three behaviors (physical activity and fruit and vegetable consumption), and impact on quality adjusted life years (QALYs).

**Methods:**

Participants (*n *= 1,629) from 23 Dutch general practices were randomized into one of four groups, which received 4 TPCs, 4 TMIs, 2 of each (combined), or no intervention (control), respectively. The self-reported outcomes, measured at baseline and 73 weeks follow-up (7 months after the last intervention component), were difference in total number of guidelines met at follow-up compared to baseline, and number of QALYs experienced over 73 weeks. The costs of implementing the intervention were estimated using a bottom-up approach.

**Results:**

At 73 weeks follow-up participants showed increased adherence with 0.62 (TPC), 0.40 (TMI), 0.50 (combined), and 0.26 (control) guidelines compared to baseline, and experienced 1.09, 1.08, 1.08, and 1.07 QALYs, respectively. The costs for the control group were considered to be zero. TMI was more expensive (€107 per person) than both the combined intervention (€80) and TPC (€57). The control condition was most cost-effective for lower ceiling ratios, while TPC had the highest probability of being most cost-effective for higher ceiling ratios (more than €160 per additional guideline met, and €2,851 for each individual QALY).

**Conclusions:**

For low society's willingness to pay, the control group was most cost-effective for the number of QALYs experienced over 73 weeks. This also applied to the increase in the number of guidelines met at lower ceiling ratios, whereas at higher ceiling ratios, TPC had a higher probability of being more cost-effective than the TMI, combined or control conditions. This also seemed to apply for QALYs experienced over 73 weeks. More research is needed on the long-term efficacy of both TPC and TMI, as well as on how to increase their cost-effectiveness.

**Trial registration:**

Dutch Trial Register NTR1068

## Background

In 2005, cardiovascular disease (CVD) was estimated to account for 30% of the 58 million deaths from all causes worldwide [1] and to cost the EU economy €169 billion, with 62% of these costs being attributed to healthcare use, 21% to productivity losses, and 17% to informal care [2]. Yet CVD can largely be prevented by modifying risk factors such as unhealthy dietary behavior and physical inactivity [1]. Evidence-based interventions targeting these behaviors are thus clearly of vital importance.

Computer tailoring and motivational interviewing (MI) have been reported as promising interventions for changing health behavior [3-5]. Computer tailoring has been defined as "a strategy intended to reach one specific person, based on characteristics that are unique to that person, related to the outcome of interest, and have been derived from and individual assessment" [6]. MI has been characterized as "a collaborative, person-centered form of guiding to elicit and strengthen motivation to change" [7]. However, few individual studies have actually compared these two types of intervention [8-10]. In research that has been undertaken to date, computer tailoring and MI did not show statistically different efficacy levels in changing absolute physical activity and fruit and vegetable consumption [8,11] or in improving guideline adherence for physical activity (≥ 5 days/week for ≥ 30 minutes/day with a moderate intensity) [10,11]. However, in improving adherence to consumption guidelines for fruit (≥ 2 servings/day) and vegetables (≥ 200 grams/day), computer tailoring appeared to be more effective than MI [11].

Economic evaluations of health behavior change interventions targeting physical activity and fruit and vegetable consumption, including computer tailoring and MI interventions do exist [8,12-14], but are still scarce [15-18]. Information on the cost-effectiveness of such interventions is needed for evidence-based decision making when it comes to the large-scale implementation of computer-tailored and MI interventions [19,20]. The North Carolina Strategies for Improving Diet, Exercise, and Screening (NC STRIDES) study [8,21] did assess the cost-effectiveness of a computer-tailored print intervention, a telephone-delivered MI intervention and a combination of the two versus no intervention, but did not report a head-to-head comparison for the cost-effectiveness of computer tailoring versus MI [8].

This study reports on the economic evaluation of tailored print communication (TPC), telephone motivational interviewing (TMI), and a combination of them in comparison with each other and with no intervention in improving the number of guidelines met for three different lifestyle behaviors, and the number of quality adjusted life years (QALY) experienced over a period of 73 weeks in older adults with and without hypertension.

## Methods

### Study design

**Figure 1:**
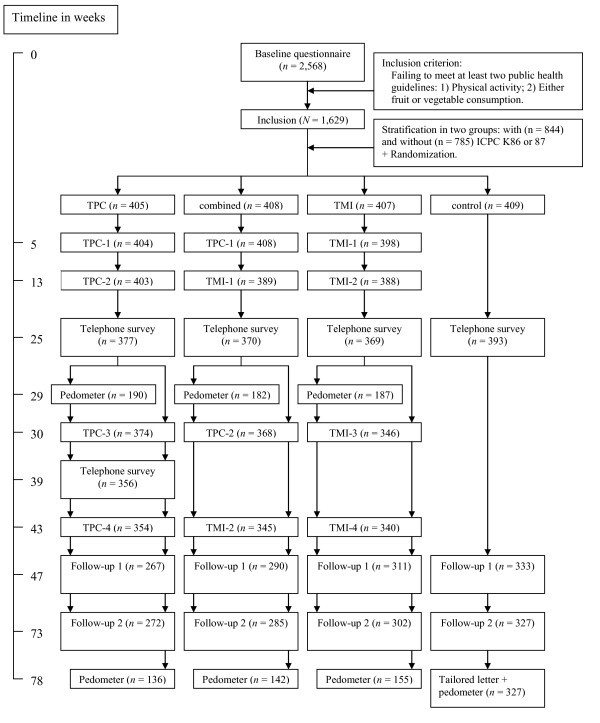
**Vitalum design and timeline**. *Notes *ICPC = international classification of primary care; K86 or K87 = hypertension without or with organ damage, respectively; TPC = tailored print communication; TMI = telephone motivational interviewing; combined = combination of TPC and TMI.

A detailed description of the Vitalum study can be found elsewhere [9]. It was approved by the Medical Ethics Committee of Maastricht University and the University Hospital Maastricht, and is registered with the Dutch Trial Register (NTR1068). Vitalum participants (*n *= 6,420) were randomly selected from 23 general practices [22,23] in two southern provinces of the Netherlands (Limburg and Noord-Brabant). The following recruitment aims were used: aged 45-70; ± 50% diagnosed by their GP as hypertensive according to the International Classification of Primary Care (ICPC code K86 or K87 for hypertension without or with organ damage respectively) [24-26]; ± 50% male; not participating in other studies according to the GP database; and only one person per address. Hypertension status was included as a recruitment aim to check whether already having a risk factor for cardiovascular disease [27] moderates the intervention effects [9]. To ensure participants' suitbility for the study, some of those selected were excluded (*n *= 875; 14%) by GPs before receiving an invitation (see for exclusion criteria [9]). After this exclusion, 5,545 participants (86% of the selection) received a Vitalum invitation from their GP explaining the content of the study and the group assignment. Those who consented to participate (*n *= 2,881) received a written baseline questionnaire. People who returned the baseline questionnaire were included in Vitalum if they failed to meet at least two out of three Dutch public health guidelines: those for physical activity and either fruit or vegetable consumption. In total, 1,629 (63%) of the 2,568 participants who filled out the baseline questionnaire were included in Vitalum. They were stratified based on their GP's diagnosis of hypertension, then the first author used a computer program to randomly link them to one of the four groups: TPC, TMI, combined or control (see Figure 1 for the study design and timeline).

### Interventions

Participants in the *TPC *group received four printed, tailored letters; the first was approximately 4 pages and addressed physical activity, the second and fourth were about 5 pages and focused on fruit and vegetables, and the third was around 3 pages and dealt again with physical activity.

Participants in the *TMI *group received four telephone calls based on MI. Participants chose the order of the conversation topics in the first and third interviews; if physical activity was preferred in the first interview, fruit and vegetable consumption was discussed in the second, and vice versa. The same procedure took place in interviews 3 and 4. Interviewers received six 3-hour training sessions from two certified trainers, after which they were required to perform one TMI conversation with adequate integrity before being appointed. Eligible interviewers were students at Maastricht University. In total, 39 out of 53 students finished the training, 34 out of 39 performed with adequate integrity, and 16 out of 34 were contracted to work on Vitalum. They used an interview protocol, received additional information (e.g. general information about CVD), and after each interview wrote a summary to assist in the next interview.

Participants in the *combined *group received two tailored print letters and two telephone motivational interviews in turns. The first tailored letter was about 4 pages; the second was approximately 5 pages. The first letter and interview focused on physical activity; the second letter and interview on fruit and vegetable consumption.

Participants in the *control *group received one tailored letter after the last follow-up questionnaire (at 73 weeks).

All tailored letters were computer-generated, i.e. participants' questionnaire answers were scanned and linked to messages filed in the computer. Hardcopy letters were mailed to participants' home addresses.

Participants from the three intervention groups received their four intervention components at 5, 13, 30, and 43 weeks after they had returned the baseline questionnaire. A telephone survey held part-way through the study (week 25) was used to assess all participants' behaviors and determinants in order to gather the most recent information for the computer-tailored intervention (i.e. the letters after week 25). Participants in the TPC group completed an additional survey (week 39) to collect the most recent data on their behavior and its determinants for the fourth tailored letter. Intervention effects were assessed using two postal questionnaires at 47 and 73 weeks follow-up.

### Effects

Because Vitalum focused on the combination of three behaviors [9] and intervention costs cannot be separated for each behavior, the cost-effectiveness analysis focused on effects on overall outcomes. Outcomes therefore included the increase in the total number of public health guidelines met from baseline to 73 weeks follow-up, and the number of QALYs experienced over 73 weeks. Choosing for guideline adherence as the outcome variable for a cost-effectiveness analysis suits with health promotion policy goals because these are often formulated in terms of proportions of people adhering to guidelines [1,28-30]. Besides guideline adherence, we chose QALYs as the outcome measure for the cost-effectiveness analysis because it has the advantage of allowing comparison of intervention effects focusing on different behaviors, domains or populations.

*Guideline adherence *was determined for physical activity and consumption of fruit and vegetables using self-report measures. The Dutch guidelines for these behaviors, which are based on international public health recommendations, were used to determine adherence. These guidelines recommend that adults consume at least two servings (approximately 200 grams in total) of fruit per day and 200 grams of vegetables per day, and engage in moderately intensive physical activity at least five days a week for 30 or more minutes a day [28,29,31-35]. Adherence to the guidelines for fruit and vegetable consumption was measured using the short questionnaire for fruit and vegetable intake [36]. Adherence to the physical activity guideline was measured using the modified CHAMPS physical activity questionnaire [37], where the total hours per week of moderately intensive physical activity was calculated with a minimum of three metabolic equivalents as cut-offs [33,38]. Added to this was the summary question ("How many days a week do you cycle, engage in do-it-yourself activities, do gardening, play a sport or engage in other strenuous physical activities for at least 30 minutes a day?") of the Short QUestionnaire to ASsess Health-enhancing physical activity (SQUASH) [39]. The reason for this addition was that the modified CHAMPS does not allow for determination of whether participants were physically active with moderate intensity for at least 5 days a week. Participants were only coded as meeting the physical activity guideline if they were physically active with at least moderate intensity for at least 2.5 hours a week according to the modified CHAMPS, and answered "five or more days" to the SQUASH summary question [29,32,33].

*Health-related quality of life *was assessed with the RAND 36-item Health Survey 1.0 [40,41]. QALYs were calculated which combined quality and quantity of life in a single measure. Quality of life is expressed as the desirability or societal preference of the patient's health state (a utility score) [42]. The SF-6 D [43] was used to estimate the utility of the health status reported by participants. QALYs were estimated by multiplying the average utility of baseline, and 47 and 73-weeks follow-up with the corresponding time, assuming a linear increase (or decrease) over time. Given this time horizon, the maximum QALY score is 1.4 (i.e. 73 weeks follow-up/52 weeks).

### Costs

Only those costs involved in implementing the intervention (e.g. printing and mailing letters for TPC, call charges for TMI) and the costs of the time invested by participants were included. Other healthcare consumption costs were expected to be equal between the groups, and were thus not measured. The developmental costs of the interventions were calculated for the number of eligible participants, and were therefore nil. Protocol-driven costs (i.e. the costs of gathering data as part of a clinical trial) were considered to be sunk costs and not included in the cost-effectiveness analysis [44].

Costs were divided into fixed costs, that are costs unrelated to the number of participants (e.g. purchase of equipment), variable costs, that are cost related to the number of participants (e.g. postal charges), and total costs, that is the sum of the fixed and variable costs. Most prices were based on actual costs, i.e. invoices. In other cases, Dutch pricing guidelines [45] were used to determine the costs. Participant time was valued €8.54 per hour based on a shadow price (i.e. lost leisure time valued equally for each participant by using the hourly wage of a legally employed domestic cleaner) as recommended by Oostenbrink et al. [45], and included time spent on participating in the intervention (e.g. reading TPCs or engaging in TMIs). With regard to MI, training costs were calculated for the number of participants who can potentially be trained per counselor. Also, the hourly wage of motivational interviewers were based on the salaries of practice assistants (€31.87) because they are most likely to be used in delivering TMI if the intervention was implemented on a large scale. An overview of the total cost of Vitalum interventions is presented in Table S1, additional file 1.

### Cost-effectiveness ratios

Incremental cost-effectiveness ratios (ICERs) were calculated by dividing the difference in costs by the differences in effects between two alternatives. Beginning with the least costly strategy, alternatives were compared with the next least costly strategy to calculate ICERs. In cases where dominance is reported, the dominating strategy is more effective and less costly than one of the other strategies. Extended dominance exists if the ICER for a given alternative is higher than that of the next, more effective alternative [44].

### Statistical analysis

Independent t-tests were executed (SPSS version 15.0; SPSS Inc, Chicago, Illinois) to examine the differences in outcomes between the groups. Selective dropout was investigated by way of mixed logistic regression using PQL estimation with MLwiN software [46].

Dropout was the dependent variable, and group, time of measurement, group by time of measurement interactions, baseline age, gender, hypertension, region and education level were used as predictors.

Cost data were complete for all groups. To account for the skewed distribution of costs, nonparametric bias-corrected accelerated bootstrapping with 2000 replications was performed using Stata to estimate percentile-based 95% confidence intervals around the differences in costs [47].

The effect data were not complete; thus, multiple imputation techniques were used to result in five complete data sets [48]. Multiple imputation is the state-of-the-art method for handling missing outcomes [49,50]. The complete cases as well as the imputed cases were then analyzed to check for bias introduced by participant dropout and missing data. The multiply imputed costs and effects were simultaneously bootstrapped in Microsoft Excel (2000 replications), and for each iteration net monetary benefits were calculated using various ceiling ratios, i.e. society's willingness to pay for the effect [51]. The strategy with the highest net monetary benefit at a specific ceiling ratio was considered most cost-effective for that specific ceiling ratio. Cost-effectiveness acceptability curves were estimated to depict visually the probability that an intervention is more cost-effective than the alternatives at various ceiling ratios. The imputed analysis showed similar results to the analyses without imputation of missing values. The results of this imputed analysis are presented below, with the complete case analyses being mentioned only where they differ from the imputed analysis.

## Results

### Sample

Mean age of the sample was 57.15 years (SD = 7.13), half of the participants (52%) were classified as hypertensive due to the inclusion criteria, 55% were men, 54% had a low (less than secondary or vocational education) and 23% had an intermediate education level (secondary through pre-university education). There were no relevant differences in demographic variables and on adherence to separate guidelines between groups at baseline (all *p *> .05).

Results of the interventions with regard to absolute behavior change revealed that TMI, TPC and the combined intervention were equally effective; participants in these groups increased their level of physical activity (hours/week) and intake of fruit (servings/day) and vegetables (grams/day) significantly more than those in the control group (Cohen's *d *effect sizes ranged from .15 to .18) [11].

As regards guideline adherence [11], none of the participants met the guideline for physical activity at baseline due to the inclusion criterion, whereas 44% and 31% of the participants adhered to the guidelines for fruit and vegetable intake, respectively. At 73 weeks follow-up, 27% (physical activity), 61% (fruit consumption) and 40% (vegetable intake) of participants in the TPC group adhered to the guidelines; this was 24%, 50% and 36% for the TMI group, respectively, 29%, 48% and 34% for the combined intervention, and 23%, 44% and 28% for the control group. For adherence to the physical activity guideline, all three interventions seemed equally effective, and the following ranking applied between groups: combined (OR compared to control = 2.08) > = TPC (OR = 1.82) > = TMI (OR = 1.57) > control (with ' > ' representing a significant difference and '> =' representing a borderline or no significant difference). With regard to adherence to the recommendations for fruit and vegetable consumption, TPC seemed to be the most effective intervention. The following ranking seemed to apply for fruit: TPC (OR compared to control = 1.78) > = TMI (OR = 1.44) > = combined (OR = 1.17) > = control. For vegetables the ranking was TPC (OR = 1.73) > = TMI (OR = 1.32) = combined (OR = 1.31) > = control.

The mean number of baseline guidelines (range 0 to 3) met was 0.8 (*sd *= 0.7) in the TPC group, 0.7 (*sd *= 0.7) in the TMI group, 0.7 (*sd *= 0.7) in the combined group, and 0.8 (*sd *= 0.7) in the control group. Baseline differences between groups in mean number of guidelines met were not significant (*p *= .52). Mean utility at baseline did not differ between groups (*p *= .48): 0.77 (*sd *= 0.11) in the TPC group, 0.76 (*sd *= 0.10) in the TMI group, 0.76 (*sd *= 0.10) in the combined group, and 0.76 (*sd *= 0.10) in the control group.

The number of dropouts was significantly higher among participants who received TPC (TPC and combined groups; weeks 25, 47, and 73: 8%, 32%, and 32%) than among participants who did not (TMI and control groups; weeks 25, 47, and 73: 6%, 22%, and 22%). Also, participants with a low education level (i.e. less than secondary or vocational education) were more likely to drop out than participants with higher education levels (25% vs. 17%). No relation was found between dropout and age, gender, hypertension, or region.

### Effects

Table 1 shows the mean values of the self-reported outcomes per group, i.e. difference in total number of public health behavior guidelines met between baseline and 73 weeks follow-up, and number of QALYs experienced over 73 weeks. Participants in the control group showed the smallest improvement in total number of guidelines met (0.3), whereas those in the TPC group showed the largest improvement (0.6). The same pattern held for the number of QALYs experienced over 73 weeks.

**Table 1:** Pooled mean values (standard errors) of the outcome variables per group in Vitalum

Outcome variable	TPC (n = 405)	TMI (n = 407)	combined (n = 408)	control (n = 409)
Difference in total number of guidelines met	0.62 (0.06)	0.40 (0.05)	0.50 (0.06)	0.26 (0.05)
Quality-Adjusted Life-Years experienced over 73 weeks	1.09 (0.01)	1.08 (0.01)	1.08 (0.01)	1.07 (0.01)

*Notes *TPC = tailored print communication; TMI = telephone motivational interviewing; combined = combination of TPC and TMI.

Group comparisons on the outcome variables are reported in Table 2. Participants in the TPC, TMI, and combined groups improved on significantly more guidelines from baseline to 73 weeks follow-up than participants in the control group. Also, participants in the TPC group did significantly better than participants in the TMI group on this outcome.

**Table 2:** Pooled mean differences (standard errors) and *p *values* of group comparisons on the outcome variables

Outcome variable	TPC vs. combined	TPC vs. TMI	TPC vs. control	combined vs. TMI	combined vs. control	TMI vs. control
Difference in total number of guidelines met	0.12 (0.07) *p *= 0.10	0.20 (0.07) *p *= 0.001	0.36 (0.07) *p *< 0.001	0.10 (0.07) *p *= 0.13	0.24 (0.07) *p *< 0.001	0.14 (0.06) *p *= 0.02
Quality-Adjusted Life-Years experienced over 73 weeks	0.01 (0.01) *p *= 0.35	0.00 (0.01) *p *= 0.73	0.02 (0.01) *p *= 0.09	-0.01 (0.01) *p *= 0.42	0.01 (0.01) *p *= 0.32	0.02 (0.01) *p *= 0.07

*Notes *TPC = tailored print communication; TMI = telephone motivational interviewing; combined = combination of TPC and TMI; *p values obtained by independent t tests

Group differences (i.e. interventions compared to no information, and interventions compared to each other) in number of QALYs experienced over 73 weeks were small and not statistically significant. In the complete case analysis, however, the difference in number of QALYs between participants in the TPC and control group, and between participants in the TMI and control group were statistically significant (*p *< .001 and *p *< .05, respectively).

### Costs

Table 3 summarizes the cost prices of the interventions as well as mean variable, fixed, and total costs per group. The costs for the control group were zero, as the participants in this group received no intervention. TPC was the least and TMI the most expensive intervention. Table 4 summarizes the group comparisons of intervention costs and shows the following ranking of costs (starting from the most expensive strategy): TMI > combined > TPC > control.

**Table 3:** Mean costs in euros (standard deviation) per participant of Vitalum groups

Costs	TPC (n = 405)	TMI (n = 407)	combined (n = 408)	control (n = 409)
Variable	40 (6)	92 (22)	65 (14)	0 (0)
Fixed	17 (0)	15 (0)	15 (0)	0 (0)
Development	0	0	0	0
Training	0	0.95	0.58	0
Implementation	16.94	13.09	13.82	0
Overhead	0.16	1.45	0.98	0

Total	57 (6)	107 (22)	80 (14)	0 (0)

*Notes *TPC = tailored print communication; TMI = telephone motivational interviewing; combined = combination of TPC and TMI.

**Table 4:** Mean differences (95% confidence intervals) and *p *values of group comparisons*

Costs	TPC vs. combined	TPC vs. TMI	TPC vs. control	combined vs. TMI	combined vs. control	TMI vs. control
Variable	-24 (-26 to -23) *p *< 0.001	-52 (-54 to -49) *p *< 0.001	40 (40 to 41) *p *< 0.001	-27 (-29 to -24) *p *< 0.001	65 (63 to 66) *p *< 0.001	92 (89 to 94) *p *< 0.001
Fixed	2	2	17	-0.1	15	15
Total	-23 (-24 to -21) *p *< 0.001	-50 (-50 to -47) *p *< 0.001	57 (57 to 58) *p *< 0.001	-27 (-30 to -25) *p *< 0.001	80 (79 to 81) *p *< 0.001	107 (105 to 109) *p *< 0.001

*Notes *TPC = tailored print communication; TMI = telephone motivational interviewing; combined = combination of TPC and TMI; *Confidence intervals and *p *values were obtained by bias corrected accelerated bootstrapping with 2000 replications in Stata.

### Cost-effectiveness analyses

Table 5 gives an overview of the ICERs. This table shows that the combined intervention and TMI are both dominated by control and TPC for the difference in total number of guidelines met. The ICER for the TPC group in comparison with the control group was €160.

**Table 5:** Incremental cost-effectiveness ratios (ICER) of Vitalum groups

Outcome	Group	Costs (euros)	Improvement in outcome	Difference with next less costly group		ICER
					
				Costs	Outcomes	
Difference in total number of guidelines met	control	0	0.26	0	0.00	
	TPC	57	0.62	57	0.36	160
	combined	80	0.50	23	-0.12	Dominated by control and TPC
	TMI	107	0.40	27	-0.10	Dominated by control and TPC

Quality-Adjusted Life-Years experienced over 73 weeks	control	0	1.07	0	0.00	
	TPC	57	1.09	57	0.02	2,867
	combined	80	1.08	23	-0.01	Dominated by control and TPC
	TMI	107	1.08	27	0.01	Dominated by extended dominance

*Note *ICER = incremental cost-effectiveness ratio (difference in costs divided by difference in effects of two groups); TPC = tailored print communication; TMI = telephone motivational interviewing; combined = combination of TPC and TMI.

For the number of QALYs experienced over 73 weeks, the combined intervention was dominated by control and TPC, while the latter two groups showed extended dominance over the TMI intervention. The ICER for the TPC group in comparison with the control group was €2,867 per QALY experienced over 73 weeks.

**Figure 2:**
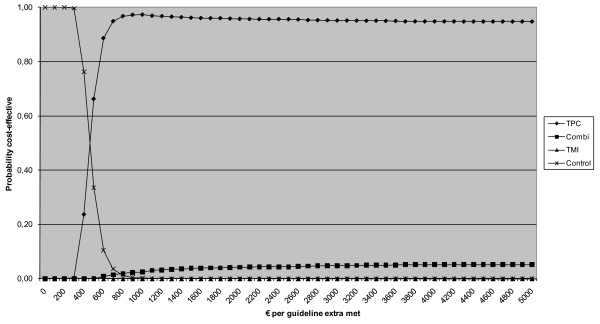
**Cost-effectiveness acceptability curve of difference in total number of guidelines met**. *Notes *TPC = tailored print communication; TMI = telephone motivational interviewing; combined = combination of TPC and TMI.

**Figure 3:**
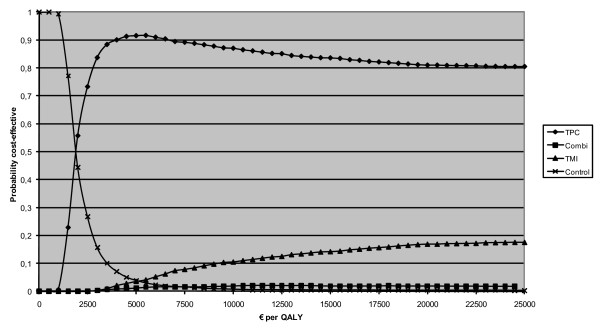
**Cost-effectiveness acceptability curves of number of Quality-adjusted life-years experienced over 73 weeks**. *Notes *TPC = tailored print communication; TMI = telephone motivational interviewing; combined = combination of TPC and TMI.

The cost-effectiveness acceptability curves of the Vitalum groups are shown for each outcome in figures 2 and 3. In no cost-effectiveness analyses were TMI or the combined intervention considered the most cost-effective option.

With regard to the difference in the total number of guidelines met at 73 weeks follow-up compared to the baseline measurement (Figure 2), the control group had the highest probability of being cost-effective for ceiling ratios below €160. For ceiling ratios higher than €160, TPC was the most cost-effective option. Similarly, for ceiling ratios of €226 or more, the probability of TPC being the most cost-effective intervention was 95% or higher.

With respect to the number of QALYs experienced over 73 weeks (Figure 3), the control group had the highest probability of being the most cost-effective for ceiling ratios lower than €2,851 per QALY. For ceiling ratios higher than €2,851, TPC seemed to be the most cost-effective strategy; however, its probability of being the most cost-effective ranged from 50% at a ceiling ratio of €2,851 up to a maximum probability of 80% at a ceiling ratio of €8,200.

## Conclusions

This article has presented an economic evaluation of the comparative cost-effectiveness of TPC, TMI, a combination of them, and no intervention in improving (i) adherence to public health guidelines for three lifestyle behaviors, and (ii) health-related quality of life in older adults with and without hypertension.

With regard to the increase in total number of guidelines met, the control group had the highest probability of being the most cost-effective at lower ceiling ratios (< €160) and the TPC group at higher ceiling ratios (> €160). For this outcome, neither the TMI nor the combined intervention were more cost-effective at any point than the TPC and control groups. Considering the large group of people who may be eligible for a TPC intervention, i.e. the population of adults aged 45-70 years in the Netherlands (5,298,716 people in 2009 [52]), widespread implementation of the TPC intervention could have an enormous impact on the Dutch healthcare budget. Thus, before decision makers can decide whether they are willing to pay this amount of money, research is needed to investigate whether the long-term benefits of TPC justify this investment.

As regards the number of QALYs experienced over 73 weeks, no intervention (i.e. the control group) was the most cost-effective strategy at lower ceiling ratios (< €2,851) and the TPC group seemed most cost-effective at higher ceiling ratios (> €2,851).

The Dutch Council for Public Health and Health Care has recently proposed thresholds for expenditures on QALYs depending on the disease burden [53], with a maximum threshold of 80,000 euro per QALY for a relative disease burden of 1.0 (maximum). Compared to the threshold of hypertension, estimated as a relative disease burden of 0.26 [53] which implies a threshold of 20,800 euro per QALY, the costs of the TPC intervention in the present study were much lower per QALY. Comparing the probability of the different strategies being the most cost-effective at higher ceiling ratios indicates that control and combination should not be considered and TPC has a four time higher probability (80% at max) of being most cost-effective compared to TMI. However, because there is no consensus regarding the minimal probability at which an intervention should be considered cost-effective compared to its alternative, results with regard to the cost per QALY need to be considered cautiously. In addition, group differences in the number of QALYs experienced over 73 weeks follow-up were very small and clinically irrelevant. Also, despite the fact that half of the participants had hypertension and they failed at least two out of three health behavior guidelines, participants valued their health as relatively good (mean baseline utility scores ranged from 0.76 to 0.77). Although QALYs have the advantage of allowing comparison of intervention effects focusing on different behaviors, domains or populations, it is difficult to find significant effects on QALYs in a relatively healthy population, such as that described here. Quality of life appears to be rather stable and most likely to change temporarily as a result of major life events, e.g. a car accident. This can be explained by the Set-Point Model, which states that everyone has a baseline quality of life level to which they will return after changes in life circumstances [54,55].

The participants' QALY scores in the present study (approximately 1.1 in all groups) were somewhat lower than the maximum QALY score of 1.4 given the time horizon. This was most likely caused by their age (45-70 years), in view of the fact that aging may be associated with functional decline and chronic disease [56,57]. Moreover, as a result of our recruitment aim, half of the participants had been diagnosed with hypertension [9].

The results of this economic evaluation differ from those of NC STRIDES [8,21]; the latter study found its combined intervention to be the most cost-effective for fruit and vegetable consumption. Participants in the combined NC STRIDES group received four TPC and four TMI components, whereas participants in the combined Vitalum group received only two of each. As is to be expected, the NC STRIDES combined group showed larger effects than the Vitalum combined group. In contrast, the effects on absolute fruit and vegetable consumption in the TPC and TMI groups in Vitalum (which for both groups were significantly greater than in the control group) were stronger than in NC STRIDES (where neither intervention significantly differed from the control group). Another explanation for the differences between Vitalum and NC STRIDES may be the outcome chosen for the cost-effectiveness analysis. The outcome of the NC STRIDES analyses was restricted to fruit and vegetable consumption, whereas that for Vitalum was the total number of guidelines met for three behaviors (physical activity as well as fruit and vegetable consumption).

TMI was the most expensive intervention due to the fact that it is delivered personally (thus the counselors' salaries and call charges need to be paid). Data entry accounted for more than half of the fixed intervention costs, with the largest share for TPC. These costs could be reduced in the future if electronic surveys are used instead. With regard to computer tailoring, the intervention costs could be further reduced by combining electronic surveys with cheaper modes of delivery, for example the internet. The cost-effectiveness of such second-generation tailored interventions should therefore be examined.

The economic evaluation presented here may overestimate the costs of TMI due to the fact that the data entry costs of the measurements were included in the cost calculations. Data were used to provide behavioral feedback at the start of the TMI sessions. Assessing an individual's health behavior by way of surveys, as carried out here, is not an essential part of MI [7], and could be replaced by a brief assessment at the start of the TMI session. Furthermore, the fidelity of MI delivery in the present study was rated as substandard for some elements [11] of the Motivational Interviewing Treatment Integrity Code 3.0 [58]. More research into the cost-effectiveness of TMI is therefore required.

Although we did not measure the cost-effectiveness of the interventions on decreased CVD risk by means of epidemiological modeling, the interventions may be expected to also decrease CVD risk, depending on how long the intervention effects are maintained. As outlined in the introduction, unhealthy dietary behavior and low levels of physical activity are modifiable risk factors of CVD [1], estimated to account for 31% (fruit and vegetable intake) and 22% (physical inactivity) of ischemic heart disease [59]. Moreover, lack of physical activity and inadequate nutrition often co-occur [60-65], which further increases the risk of morbidity, mortality and healthcare costs [66,67]. We therefore recommend that future cost-effectiveness studies targeting CVD risk behaviors either employ a longer follow-up period to examine how CVD risk is affected, or use modeling techniques.

A limitation of the present study is the use of self-reporting measures in assessing guideline adherence, as self-reports are prone to measurement bias such as socially desirable answers [68-70]. Another limitation is the somewhat restrictive perspective used for the economic evaluation, which may have led to the exclusion of important costs from the societal perspective [71], for example changes in productivity and healthcare utilization [72]. Although an analysis from a societal perspective would provide the most complete information for decision makers [71], we were unable to take this angle as our time frame was restricted to 73 weeks follow-up and we did not expect any effect on overall healthcare utilization or lost productivity during this period. Also, we wanted to reduce the participant burden by excluding questions about healthcare use. A third limitation was that dropout was higher in participants with a low education level than with higher levels of education. The cost-effectiveness analyses could be biased in case of non-ignorable dropout (i.e. dropout depending on unmeasured outcome variables). Because the cost-effectiveness analyses were intention-to-treat [50], and dropout did not seem to depend on other covariates, the analyses were unbiased under the assumption of missingness at random. In addition, dropout rates were highest among participants receiving TPC and the combined intervention. This could be explained by the participant evaluation of the interventions [11]. Participants who received TMI were more satisfied with the intervention and perceived it as more interesting than did participants who received TPC. Another reason could be the personal delivery of TMI, as this may enhance commitment among participants. Finally, a disadvantage of using overall outcomes in stead of focusing on separate behavioral outcomes in the cost-effectiveness analysis is that the difference in effect between the behaviors are neglected and valued equally. However, as was described before, the cost-effectiveness in the present study was assessed for overall outcomes because Vitalum focused on the combination of three behaviors [9] and intervention costs could not be separated for each behavior.

Overall, this economic evaluation indicated that the control group, i.e. the group that received no intervention, displayed the most cost-efficacy for the number of QALYs experienced over 73 weeks when willingness to pay by the society was low. This also applied to the increase in the number of guidelines met at lower ceiling ratios, whereas at higher ceiling ratios, TPC had a higher probability of being more cost-effective than TMI, the combined version or control. With regard to QALYs experienced over 73 weeks, the TPC intervention seemed most cost-effective at higher ceiling ratios. Future research should examine the long-term effectiveness of the interventions as well as strategies to increase the cost-effectiveness of TPC and TMI.

## Competing interests

The authors declare that they have no competing interests.

## Authors' contributions

IM and JB obtained the finances for this study. HMVK and IM developed and executed the study, with HDV advising on its development. JLS advised on the conceptions of data collection, analyses and execution. JEB contributed statistical expertise, and performed the analyses under the supervision of MWVT. HMVK and JEB significantly contributed to the writing of the manuscript, while IM, HDV, JB, MWVT and JLS were involved in revising it. All authors have seen and approved of the version to be published.

## Supplementary Material

Additional file 1 **Total fixed costs (in euros) per intervention group in Vitalum**. *Notes *TPC = tailored print communication; TMI = telephone motivational interviewing; combined = combination of TPC and TMI. Click here for file
