# Cost-effectiveness of tailored print communication, telephone motivational interviewing, and a combination of the two: results of an economic evaluation alongside the Vitalum randomized controlled trial

**DOI:** 10.1186/1479-5868-8-4

**Published:** 2011-01-26

**Authors:** Hilde M van Keulen, Judith E Bosmans, Maurits W van Tulder, Johan L Severens, Hein de Vries, Johannes Brug, Ilse Mesters

**Affiliations:** 1 School for Public Health and Primary Care (Caphri), Department of Health Promotion, Maastricht University, the Netherlands; 2 Department of Health Sciences and EMGO Institute for Health and Care Research, VU University Amsterdam, the Netherlands; 3 Institute for Health Policy and Management, Erasmus University Rotterdam, the Netherlands & School for Public Health and Primary Care (Caphri), Department of Health Organization, Policy, and Economics, Maastricht University, Maastricht, the Netherlands; 4 EMGO Institute for Health and Care Research, VU University Medical Centre, Amsterdam, the Netherlands; 5 School for Public Health and Primary Care (Caphri), Department of Epidemiology, Maastricht University, the Netherlands

## Correction

Since publication of our article [1], we have realized that Figure two (Figure 1): "Cost-effectiveness acceptability curve of difference in total number of guidelines met", is incorrect. The original text referring to the figure as well as the figure legend is correct. The correct Figure two (Figure 1) is displayed here. We apologise for any inconvenience or confusion this may have caused.

**Figure 1:**
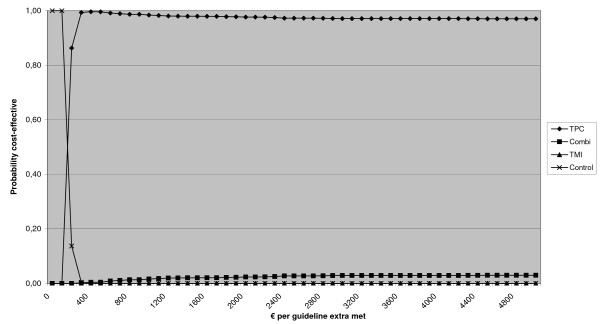
**Cost-effectiveness acceptability curve of difference in total number of guidelines met**. *Notes *TPC = tailored print communication; TMI = telephone motivational interviewing; combined = combination of TPC and TMI.
