# Supplementary material for: Cost-effectiveness of tailored print communication, telephone motivational interviewing, and a combination of the two: results of an economic evaluation alongside the Vitalum randomized controlled trial
Source: Int J Behav Nutr Phys Act. 2010 Sep 3;7:64. doi: 10.1186/1479-5868-7-64 (PMC2940922; doi:10.1186/1479-5868-7-64)
Supplement: Additional file 1 — Total fixed costs (in euros) per intervention group in Vitalum. Notes TPC = tailored print communication; TMI = telephone motivational interviewing; combined = combination of TPC and TMI. [file 1479-5868-7-64-S1.DOC]

**Table S1 -** Total fixed costs (in euros) per intervention group in Vitalum

| **Type of costs** | **Item** | **TPC** | **TMI** | **Combined** |
| --- | --- | --- | --- | --- |
| Development | Training MI | 0 | 2,728 | 2,728 |
|  | TPC | 25,322 | 0 | 25,322 |
|  | Measurements | 2,679 | 1,264 | 2,529 |
|  | MI protocol | 0 | 3,358 | 3,358 |
|  | Total | 28,000 | 7,350 | 33,937 |
| Training | Trainers | 0 | 1,959 | 1,959 |
|  | Interviewers | 0 | 20,888 | 20,888 |
|  | Total | 0 | 11,424 | 11,424 |
| Implementation | TMI | 0 | 3,307 | 3,307 |
|  | TPC | 10,102 | 0 | 10,102 |
|  | Data entry | 17,951 | 12,794 | 12,794 |
|  | Total | 28,053 | 16,101 | 26,202 |
| Overhead | Rental costs | 94 | 376 | 470 |
|  | Cleaning costs | 17 | 70 | 87 |
|  | Service costs | 410 | 650 | 650 |
|  | Total | 521 | 1,096 | 1,207 |
| **Total** |  | **56,574** | **35,971** | **72,770** |

*Notes* TPC = tailored print communication; TMI = telephone motivational interviewing; combined = combination of TPC and TMI.
